# Increasing Burden of Early-Onset Cancers: Disentangling the Contributions of Changes in Risk from Demographic Shifts

**DOI:** 10.1158/2767-9764.CRC-26-0176

**Published:** 2026-07-01

**Authors:** Axelle Braggion, Bernadette Wilhelmina Antonia van der Linden, Stefano Tancredi, Sabine Rohrmann, Stéphane Cullati, Arnaud Chiolero

**Affiliations:** 1 Population Health Laboratory (#PopHealthLab), University of Fribourg, Fribourg, Switzerland.; 2 Swiss School of Public Health (SSPH+), Zurich, Switzerland.; 3 Swiss Center of Expertise in Life Course Research LIVES, Lausanne, Switzerland.; 4 Cancer Registry of the Cantons of Zurich, Zug, Schaffhausen, and Schwyz, University Hospital Zurich, Zurich, Switzerland.; 5 Epidemiology, Biostatistics and Prevention Institute (EBPI), University of Zurich, Zurich, Switzerland.; 6 School of Population and Global Health, McGill University, Montreal, Canada.

## Abstract

The burden of early-onset cancers is increasing, attracting scientific and media attention. There is, however, a need to disentangle the contribution of changes in risk, that is, age-standardized rate, due to changes in carcinogenic exposure or detection practices, from the contribution of population growth and aging, and to determine how these contributions differ for later-onset cancers. Using cancer registry data, we estimated these contributions in Switzerland between 1982 and 2021. Early-onset cancers include any cancer diagnosed in adults between 20 and 49 years, whereas later-onset cancers are those diagnosed at 50 years or older. To estimate the contribution of changes in risk, we computed the expected number of cases by applying the 1982 to 1986 (baseline) age-standardized rates to subsequent populations and calculated the difference between observed and expected cases. To estimate the contribution of population growth and aging, we subtracted the risk contribution from the total change in observed cases relative to 1982 to 1986. Between 1982 to 1986 and 2017 to 2021, the burden of early-onset cancers increased by 1,534 cases/year and that of later-onset cancers increased by 19,832. For early-onset cancers, population growth and aging explained 71% of the increase (men: 102%; women: 58%) and changes in risk explained the remaining 29% (men: −2%; women: 42%). These proportions differ by cancer site, with risk contributing to a higher proportion for prostate cancer, colorectal cancer, and melanoma. For later-onset cancers, population growth and aging explained 92% of the increase (men: 116%; women: 81%) and changes in risk explained the remaining 8% (men: −16%; women: 19%). Further research is needed to clarify whether risk changes are due to changes in carcinogenic exposure or in detection practices.

**Significance:**

Although population growth and aging are known to contribute to increasing cancer burden, their relative contribution compared with risk changes has not been quantified for early-onset cancers. Using four decades of Swiss cancer registry data, we show that demographics explain about three quarters of the increase in early-onset cases, whereas risk changes account for the remaining quarter. Contributions vary by cancer site and sex, with implications for prevention and healthcare planning.

## Introduction

The growing burden of early-onset cancers, defined as cancers diagnosed before 50 years, has attracted considerable scientific and media attention (1–6). This increase is part of a broader increase in the overall cancer burden across all ages, with nearly 20 million new cases worldwide in 2022 compared with 11 million in 2002 (7, 8). Demographic changes, namely, population growth and aging, are key drivers of this global increase, as larger and older populations experience more cancer cases (8, 9). It is, however, underappreciated that population growth and aging may also explain a large share of the increase in early-onset cancers.

The burden of cancer, defined here as the number of new cases, is a function of the size and age structure of the population studied, as well as the risk of being diagnosed with cancer at a given age. This risk is estimated using age-standardized incidence rates and is influenced primarily by exposure to carcinogenic factors and secondarily by detection practices (6, 8, 10, 11). On the one hand, changes in carcinogenic exposure and others factors, such as the increasing prevalence of obesity, could explain the increased risk of several types of cancers in the population (6, 8, 10). Another example is smoking, in which changes in prevalence patterns closely parallel trends in lung cancer incidence (12, 13). Environmental exposures, including air pollution, are also suspected contributors to the increasing cancer burden (1, 2). On the other hand, changes in detection practices, such as advances in diagnostic test sensitivity, shifts in classification criteria, incidental findings, and broader screening guidelines, might lead to increased diagnostic scrutiny in younger and older adults, with a major and rapid effect on the incidence of so-called scrutiny-dependent cancers, e.g., prostate and thyroid cancers or melanoma (6, 8, 10, 11, 14).

How changes in demographics and risk factors influence the burden of early-onset cancers carries major and distinct implications for public health (1–4, 6, 15, 16). There is no public health measure to influence a demographic shift, yet it can have a major effect on the number of cases and direct implications for healthcare planning. Increasing risk, on the other hand, suggests the need for strengthening prevention and investigating emerging causes early in life (17). Decomposing the observed increase in the number of new cases into demographic and risk contributions is therefore essential to inform appropriate public health responses, as these contributions require distinct interventions and cannot be estimated by age-specific analysis alone. Understanding these changes also requires assessing whether the contributions of demographics and risk factors, previously examined for all cancer sites combined (18), are specific to early-onset cancers or also observed in later-onset cancers. We therefore aimed to estimate the relative contributions of changes in risk and of population growth and aging to the burden of early-onset cancers—defined as the number of new cases—between 1982 and 2021 in Switzerland, by cancer site and to compare these contributions with those of later-onset cancers.

## Materials and Methods

### Study design and setting

This study was conducted in Switzerland, a high-income country in the center of Europe with 9 million inhabitants and a life expectancy of 83.5 years, one of the highest in the world (19). In Switzerland, cancer is the leading cause of death between 45 and 84 years and around 48,000 new cases are diagnosed each year (20, 21). The five most common cancer sites include the prostate, breast, colorectum, lung, and skin. Further information on the cancer burden in Switzerland and main risk factors can be found on the Swiss Federal Statistical Office (SFSO) website and in the latest cancer report (20–22).

For this descriptive study, we followed the REporting of studies Conducted using Observational Routinely collected Data (RECORD) checklist (Supplementary Table S1; refs. 23, 24). We retrieved data from population-based registries in Switzerland including cancer cases diagnosed between January 1, 1982, and December 31, 2021. We used aggregated cancer data extracted from the Swiss national dataset managed by the National Institute for Cancer Epidemiology and Registration (NICER; ref. 25). In Switzerland, adult cancer registration is organized at the cantonal level. Each new cancer diagnosis in adults 20 years or older is reported to the cantonal cancer registry, which collects the data and transmits them to NICER, and since 2020 to the National Agency for Cancer Registration. Cancer registration coverage increased over time, reaching 97% of the Swiss population in 2017 to 2021 (26, 27). In earlier years, national cancer statistics were estimated from existing registries to extrapolate the expected data for areas not covered by a registry (27, 28). The estimates were computed by applying extrapolation weights (observed population with registration/total population), calculated for different age, sex, and linguistic region strata, to observed case counts. A recent report showed high data quality for Swiss cancer registries (29). Cancer incidence data were coded according to the 10th revision of the International Classification of Diseases (ICD-10) and included all malignant primary diagnoses (C00–C97), apart from nonmelanoma skin cancer (C44). Primary tumors were defined according to International Agency for Research on Cancer criteria.

### Study population

We included all cancer cases of permanent residents 20 years or older in Switzerland from January 1, 1982, to December 31, 2021. In addition to the main analysis of all cancers (C00–C43 and C45–C97), we conducted separate analyses for the most common cancer sites in the population: breast (C50), prostate (C61), lung/bronchus/trachea (C33–34), colorectum (C18–20), and skin (C43).

### Definition of early- and later-onset cancers

We defined early-onset cancer as any cancer diagnosed between 20 and 49 years and later-onset cancer as any cancer diagnosed at 50 years or older.

### Statistical analysis

We reported absolute numbers of new cases and age-standardized (2013 European Standard Population; ref. 30) incidence rates per 100,000 people. We conducted stratified analyses by early onset or later onset, sex (male or female), 5-year periods (1982–1986, 1987–1991, 1992–1996, 1997–2001, 2002–2006, 2007–2011, 2012–2016, or 2017–2021), and cancer sites (all sites combined, breast, prostate, lung/bronchus/trachea, colorectum, or skin).

To estimate the contribution of risk (*C*_*risk*_) to the net changes in the absolute number of cases, we first computed the expected number of cases by applying the 1982 to 1986 age-standardized rates to the population structure of each subsequent period. This uses the 1982 to 1986 rate as a counterfactual reference, representing the number of cases that would have occurred had risk remained stable from the baseline period. Any deviation between observed and expected counts therefore represents excess cases attributable to changes in risk over time.

Second, we calculated the difference between the observed and expected number of cases for each period. To estimate the contribution of population growth and aging (*C*_*demographic*_), we subtracted the risk contribution (*C*_*risk*_) from the total net change in the number of observed cases between 1982 to 1986 and each subsequent period. *C*_*demographic*_ therefore represents the excess number of cases attributable to changes in population size and age structure over time. Finally, we expressed *C*_*demographic*_ and *C*_*risk*_ as proportions of the total net change, such that *C*_*demographic*_ + *C*_*risk*_ = +100% if there is an increase in the net change and −100% if there is a decrease in the net change. Each of these proportions can be positive or negative. A positive proportion indicates that the contribution acts in the same direction as the net change in the burden. For example, if the burden keeps increasing and the contribution from population growth and aging is positive, demographic changes are the primary drivers of this increase. A negative proportion indicates that the contribution acts in the opposite direction of the net change, partially offsetting the increase or decrease in burden. Analyses were performed using R, version 4.3.2 (RRID: SCR_001905) and Excel (RRID: SCR_016137).

### Ethical approval and informed consent

We used deidentified and aggregated data, ensuring no risk to individual patient confidentiality. Until 2019, cancer registration in Switzerland operated under presumed consent and was governed by cantonal decisions. Since 2020, cancer registration has been mandated nationwide under the Swiss Cancer Registration Act (Loi fédérale sur l'enregistrement des maladies oncologiques; RS 818.33), which provides a legal basis for the systematic collection of cancer data throughout Switzerland. According to this law, diagnosing physicians are obligated to inform patients about the registration of their data and patients can refuse registration.

## Results

Details about age-standardized incidence rates can be found in Supplementary Figs. S1–S3 and have also been discussed in the previous work (18). In the present study, we focus on the contributions of changes in risk and of population growth and aging to the burden of new cancer cases.

### All cancers combined

**Figure 1.**
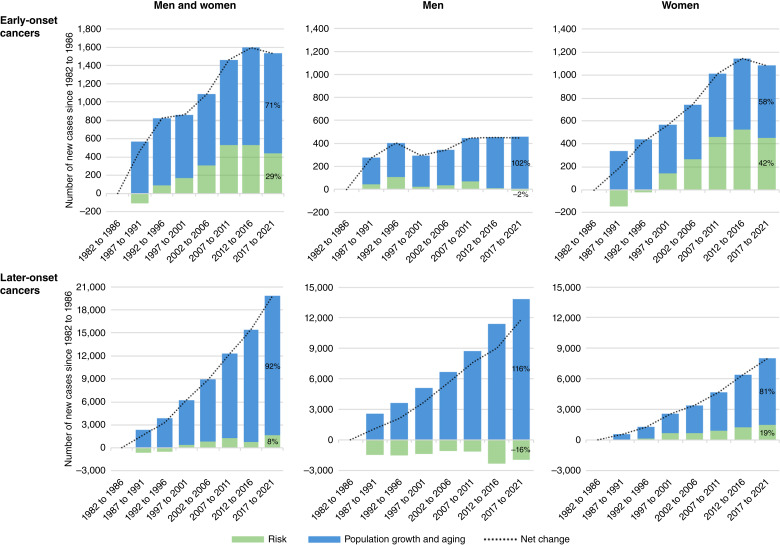
Contributions of population growth and aging and of risk to the net changes in the number of new cases of early- and later-onset cancers, all cancers combined (C00–43 and C45–97), by sex, 1982–2021, Switzerland. Dotted line shows the difference in the annual number of new cases compared with the period 1982–1986.

Between 1982 to 1986 and 2017 to 2021, the annual number of early-onset cancers increased from 3,176 to 4,709 (+48%) and of later-onset cancers from 21,870 to 41,702 (+91%; Supplementary Table S2). The burden increase was mainly due to population growth and aging for both early- and later-onset cancers (Fig. 1). Comparing 2017 to 2021 with 1982 to 1986, population growth and aging explained 71% of the increase in early-onset cancers (men: 102%; women: 58%) and risk changes accounted for 29% (men: −2%; women: 42%). For later-onset cancers, population growth and aging explained 92% of the increase (men: 116%; women: 81%) and risk changes accounted for 8% (men: −16%; women: 19%). Between 1982 to 1986 and 2017 to 2021, the proportional contribution of risk to early-onset cancer decreased in men and increased in women.

### Prostate cancer

**Figure 2.**
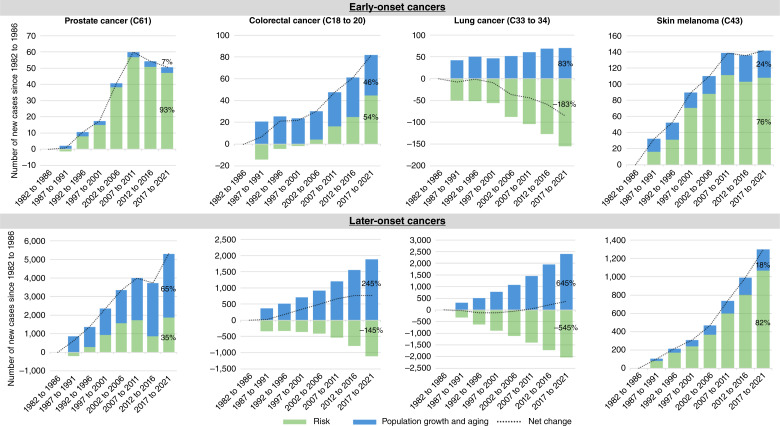
Contributions of population growth and aging and of risk to the changes in the number of new cases of early- and later-onset cancers among men, by main cancer sites (prostate, colorectum, lung, and skin), 1982–2021, Switzerland. Dotted line shows the difference in the annual number of new cases compared with the period 1982–1986.

Between 1982 to 1986 and 2017 to 2021, the annual number of early-onset prostate cancer increased from 10 to 58 (+500%) and of later-onset prostate cancer from 2,459 to 7,762 (+216%; Supplementary Table S2). Since 1982 to 1986, 7% of the increase in the burden of early-onset prostate cancer was attributable to population growth and aging, whereas 93% was attributable to increasing risk; these percentages were 65% and 35% for later-onset prostate cancer, respectively (Fig. 2). Between 1982 to 1986 and 2017 to 2021, the proportional contribution of risk increased for early-onset prostate cancer and fluctuated for later-onset prostate cancer.

### Breast cancer

**Figure 3.**
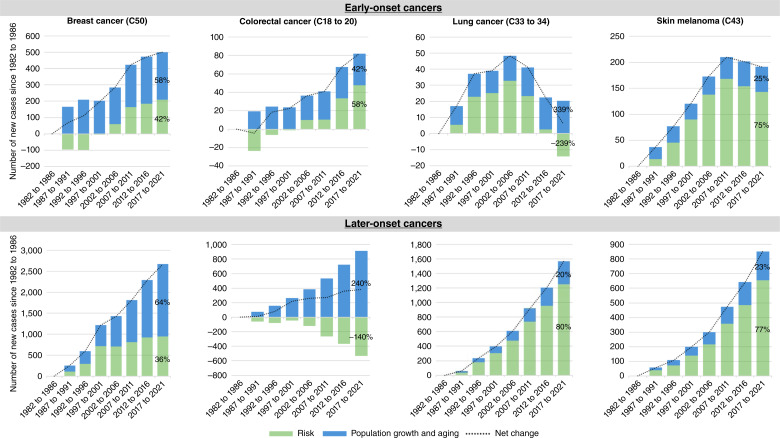
Contributions of population growth and aging and of risk to the changes in the number of new cases of early- and later-onset cancers among women, by main cancer sites (breast, colorectum, lung, and skin), 1982–2021, Switzerland. Dotted line shows the difference in the annual number of new cases compared with the period 1982–1986.

Between 1982 to 1986 and 2017 to 2021, the annual number of early-onset breast cancer increased from 764 to 1,266 (+66%) and of later-onset breast cancer from 2,677 to 5,349 (+100%; Supplementary Table S2). Since 1982 to 1986, 58% of the increase in the burden of early-onset breast cancer was attributable to population growth and aging, whereas 42% was attributable to increasing risk; these percentages were 64% and 36% for later-onset breast cancer, respectively (Fig. 3). Until 1997 to 2001, the risk decreased and mitigated the increase in early-onset breast cancer incidence but increased thereafter. For later-onset breast cancer, the contribution of population growth and aging slightly increased over time.

### Colorectal cancer

Between 1982 to 1986 and 2017 to 2021, the annual number of early-onset colorectal cancer increased from 96 to 178 (+85%) in men and from 91 to 173 (+90%) in women, whereas the annual number of later-onset colorectal cancer increased from 1,562 to 2,329 (+49%) in men and from 1,425 to 1,805 (+27%) in women (Supplementary Table S2). Since 1982 to 1986, in men 46% of the increase in the burden of early-onset colorectal cancer was attributable to population growth and aging, whereas 54% was attributable to increasing risk; these percentages were 245% and −145% for later-onset colorectal cancer, respectively (Fig. 2). Among women, 42% of the increase in the burden of early-onset colorectal cancer was attributable to population growth and aging, whereas 58% was attributable to increasing risk; these percentages were 240% and −140% for later-onset colorectal cancer, respectively (Fig. 3). Until 1997 to 2001, the risk decreased and mitigated the increase in early-onset colorectal cancer incidence among men but increased thereafter. For later-onset, the decreasing risk consistently mitigated the increase during the whole study period. Similar trends were observed among women.

### Lung cancer

Between 1982 to 1986 and 2017 to 2021, the annual number of early-onset lung cancer decreased from 158 to 73 (−54%) in men and increased from 53 to 59 (+11%) in women, whereas the annual number of later-onset lung cancer increased from 2,380 to 2,753 (+16%) in men and from 496 to 2,065 (+317%) in women (Supplementary Table S2). Since 1982 to 1986, in men 183% of the decrease in the burden of early-onset lung cancer was attributable to reduction in risk, whereas population growth and aging mitigated this decrease by 83% (Fig. 2). For later-onset lung cancer in men, 645% of the increase was attributable to population growth and aging, whereas reduction in risk mitigated this increase by 545%. Among women, 339% of the increase in the burden of early-onset lung cancer was attributable to population growth and aging, whereas risk reduction mitigated this increase by 239% (Fig. 3). For later-onset lung cancer in women, 20% of the increase was attributable to population growth and aging and 80% to higher risk. For later-onset lung cancer in men, the decreasing risk consistently mitigated the increase in the burden. Among women, early-onset lung cancer initially increased due to increasing risk and then sharply declined after 2007 to 2011 as risk decreased. For later-onset cases, the increase was mainly due to increasing risk.

### Skin melanoma

Between 1982 to 1986 and 2017 to 2021, the annual number of early-onset skin melanoma increased from 108 to 250 (+131%) in men and from 162 to 353 (+118%) in women, whereas the annual number of later-onset skin melanoma increased from 241 to 1,538 (+540%) in men and from 303 to 1,155 (+282%) in women (Supplementary Table S2). Since 1982 to 1986, 24% of the increase in the burden of early-onset skin melanoma in men was attributable to population growth and aging, whereas 76% was attributable to increasing risk; these percentages were 18% and 82% for later-onset skin melanoma, respectively (Fig. 2). Among women, 25% of the increase in the burden of early-onset skin melanoma was attributable to population growth and aging, whereas 75% was attributable to higher risk; these percentages were 23% and 77% for later-onset skin melanoma, respectively (Fig. 3). For early-onset skin melanoma, although the contribution of risk increased over time, the burden initially increased and from 2007 to 2011 onward, it stabilized in men and decreased in women. For later-onset skin melanoma, the proportional contribution of risk was stable for both men and women.

## Discussion

The increasing burden of early-onset cancers, defined as the number of new cancer cases, is shaped by two distinct components: first, demographic changes, namely, population growth and aging, and second, changes in risk, reflected in age-standardized rates and driven by both carcinogenic exposure and detection practices. The relative contributions of these two components to the burden need to be clarified. In Switzerland, in the last four decades, population growth and aging accounted for the largest share of the increase across all cancer sites combined. Population growth and aging were the main contributors to the increase in early-onset breast and lung cancers among women, whereas increasing risk contributed to a larger share of the increase in early-onset prostate cancer, colorectal cancer, and skin melanoma among men and in colorectal cancer and skin melanoma among women. Conversely, risk reduction was the main driver of the decrease in lung cancer among men. In later-onset cancers, population growth and aging were the main contributors to the increase across all cancer sites, except for skin melanoma and lung cancer in women.

Previous research has explored the impact of demographic shifts on selected early-onset cancers. For instance, in a study analyzing the burden of breast cancer among women 15 to 39 years, the authors reported that the global increase in Disability Adjusted Life Year was primarily due to population growth, followed by population aging (31). In Switzerland, the early-onset age stratum has aged in the past decades, with proportionally more individuals in 2021 in their 40s (35% of the 20–49 age group) than in their 20s (29%), compared with the 1980s (30% and 35%, respectively; Supplementary Fig. S4; ref. 32). As cancer risk increases with age, the aging of the early-onset population over the study period (1982–2021) results in a higher number of cases within this age range and thus an increase in the early-onset cancer burden (33). This shift in age structure, along with population growth, explains most of the increase in the burden of overall early-onset cancers.

Beyond demographics, the increased risk observed in early-onset colorectal cancer—but not in later-onset disease—has also been reported in recent studies (34, 35). Part of this increase may be attributable to changes in early-life exposures such as obesity (17), though evolving diagnostic classification of colorectal tumors may also play a role (35, 36). The decreased risk noted in later-onset colorectal cancer may reflect differences in carcinogenic exposure, as well as the removal of precancerous lesions among adults 50 years and older through colonoscopy screening. Similarly, sex- and age-specific trends in the risk contribution to lung cancer are likely due to divergent historical smoking prevalence patterns (12, 13). Another example is prostate cancer, in which fluctuations in risk contribution may be explained by temporal changes in prostate-specific antigen (PSA) screening practices and opportunistic screening, also among men younger than 50 years (37–41). The absolute risk among adults younger than 50 years, however, remains low for these cancer sites, as shown by age-standardized rates (colorectal cancer: 7/100,000 in 1982–1986 to 10/100,000 in 2017–2021; prostate cancer: 1/100,000 in 1982–1986 to 3/100,000 in 2017–2021; Supplementary Table S2).

A major limitation of our study is the inability to fully understand the contribution of risk, as the roles of carcinogenic exposures versus detection practices remain uncertain. The term “risk” itself is potentially misleading, as it conflates not only the effect of risk factors, namely, carcinogenic exposures, but also the effect of detection practices. Furthermore, cancer registries were gradually implemented across Swiss cantons from 1969 onward, meaning that national estimates relied on extrapolations from existing data, and some inaccuracy—particularly in earlier years—cannot be excluded (42). Strengths include that this is the first national study to estimate the contributions of population growth and aging versus changes in risk, providing insights into the growing burden of early-onset cancers. Our results may be transportable, at least partly, to countries with similar demographic dynamics and cancer detection practices (43).

Our findings highlight the importance of distinguishing the relative contributions of demographic transition from increasing risk when analyzing the early-onset cancer burden over time. If the risk is not increasing, there would be little reason for heightened concern or for further research to identify emerging early-life causes (44). Instead, the substantial contribution of population growth and aging to the growing burden of early-onset cancers shows the importance of adequate healthcare planning for the coming decades. Furthermore, the higher risk noted for cancer sites such as the prostate, colorectum, and skin needs to be interpreted cautiously. This risk may reflect changes in detection practices or classification criteria rather than a true increase in carcinogenic exposures (Supplementary Fig. S5; refs. 6, 8, 10, 11). Advances in medical tests, expanded use of screening, incidental findings, and increased awareness could result in greater detection, rather than a genuine increase in cancers (11, 45). In addition, these changes can also lead to earlier detection, shifting cases that would previously have been identified at later ages into the early-onset category (11, 45).

### Conclusions

Population growth and aging are major contributors to the increasing burden of early-onset cancers, whereas increases in risk predominantly explain the increase in prostate cancer, colorectal cancer, and skin melanoma cases. Further research is needed to clarify the extent to which the increase in risk reflects changes in carcinogenic exposures or in detection practices, as this distinction is essential for guiding public health policy and intervention strategies.

## Supplementary Material

Supplementary Table 1 The RECORD statement – checklist of items, extended from the STROBE statement, that should be reported in observational studies using routinely collected health data.

Supplementary Table 2 Mean annual absolute number of new cases, crude and age-standardized (2013 European standard) incidence rates per 100'000, by age group (early- vs later-onset), sex, 5-year period, and cancer sites, 1982-1986 vs 2017-2021, Switzerland.

Supplementary Figure 1 Mean annual age-standardized (2013 European standard) incidence rates for all cancers (C00-43, C45-97), by age group (early- vs later-onset), sex, and 5-year period, 1982-2021, Switzerland.

Supplementary Figure 2 Mean annual age-standardized (2013 European standard) incidence rates among men, by main cancer sites (prostate, colorectal, lung, skin melanoma) and age group (early- vs later-onset), and 5-year period, 1982-2021, Switzerland.

Supplementary Figure 3 Mean annual age-standardized (2013 European standard) incidence rates among women, by main cancer sites (breast, colorectal, lung, skin melanoma) and age group (early- vs later-onset), and 5-year period, 1982-2021, Switzerland.

Supplementary Figure 4 Proportion of each age subgroup (20-29, 30-39, 40-49) within the early-onset population, 1982-2021, Switzerland.

Supplementary Figure 5 Cancer burden function. The cancer burden can be depicted as a function of demographic factors (that is, population growth and ageing) on the one hand and of cancer risk on the other hand. In our study, population growth and ageing determine Cdemographic, while exposure to carcinogenic factors, detection practices, and tumors classification determine Crisk.

## Data Availability

All data used in this work are held by NICER and SFSO. The contracts with these entities preclude the direct sharing of the original data by the authors. Data can be obtained through direct requests to NICER for cancer registration data and to SFSO for mortality data.
