# Supplementary material for: Increasing Burden of Early-Onset Cancers: Disentangling the Contributions of Changes in Risk from Demographic Shifts
Source: Cancer Res Commun. 2026 Jul 1;6(7):1539–45. doi: 10.1158/2767-9764.CRC-26-0176 (PMC13319521; doi:10.1158/2767-9764.CRC-26-0176)
Supplement: Supplementary Table 2 — Mean annual absolute number of new cases, crude and age-standardized (2013 European standard) incidence rates per 100'000, by age group (early- vs later-onset), sex, 5-year period, and cancer sites, 1982-1986 vs 2017-2021, Switzerland. [file crc-26-0176_supplementary_table_2_suppst2.docx]

**Supplementary Table 2 - Mean annual absolute number of new cases, crude and age-standardized (2013 European standard) incidence rates per 100'000, by age group (early- vs later-onset), sex, 5-year period, and cancer sites, 1982-1986 vs 2017-2021, Switzerland.**

| **Incidence** | **Early-onset cancers** | | | | | | **Later-onset cancers** | | | | | |
| --- | --- | --- | --- | --- | --- | --- | --- | --- | --- | --- | --- | --- |
|  | New cases (N) | Crude rate per 100'000 | Age-std rate per 100'000 | New cases (N) | Crude rate per 100'000 | Age-std rate per 100'000 | New cases (N) | Crude rate per 100'000 | Age-std rate per 100'000 | New cases (N) | Crude rate per 100'000 | Age-std rate per 100'000 |
|  |  | **1982-1986** |  |  | **2017-2021** |  |  | **1982-1986** |  |  | **2017-2021** |  |
| **All cancers combined*** (C00-43, C45-97) | | | |  |  |  |  |  |  |  |  |  |
| Men and women | 3176 | 112 | 123 | 4709 | 135 | 136 | 21870 | 1123 | 1176 | 41702 | 1232 | 1268 |
| Men | 1365 | 96 | 104 | 1814 | 103 | 103 | 11810 | 1373 | 1576 | 23655 | 1467 | 1579 |
| Women | 1811 | 129 | 142 | 2896 | 169 | 169 | 10060 | 926 | 927 | 18047 | 1018 | 1014 |
| **Breast** (C50) |  |  |  |  |  |  |  |  |  |  |  |  |
| Women | 764 | 54 | 62 | 1266 | 74 | 74 | 2677 | 246 | 246 | 5349 | 302 | 303 |
| **Prostate** (C61) |  |  |  |  |  |  |  |  |  |  |  |  |
| Men | 10 | 0,7 | 0,8 | 58 | 3 | 3 | 2459 | 286 | 363 | 7762 | 481 | 518 |
| **Colon, rectum** (C18-20) |  |  |  |  |  |  |  |  |  |  |  |  |
| Men and women | 186 | 7 | 7 | 350 | 10 | 10 | 2987 | 153 | 163 | 4134 | 122 | 125 |
| Men | 96 | 7 | 8 | 178 | 10 | 10 | 1562 | 182 | 212 | 2329 | 145 | 156 |
| Women | 91 | 6 | 7 | 173 | 10 | 10 | 1425 | 131 | 131 | 1805 | 102 | 100 |
| **Lung, bronchus, trachea** (C33-34) | |  |  |  |  |  |  |  |  |  |  |  |
| Men and women | 211 | 7 | 9 | 132 | 4 | 4 | 2876 | 148 | 150 | 4819 | 142 | 148 |
| Men | 158 | 11 | 13 | 73 | 4 | 4 | 2380 | 277 | 295 | 2753 | 171 | 184 |
| Women | 53 | 4 | 4 | 59 | 3 | 3 | 496 | 46 | 46 | 2065 | 116 | 119 |
| **Skin melanoma** (C43) |  |  |  |  |  |  |  |  |  |  |  |  |
| Men and women | 270 | 10 | 10 | 603 | 17 | 17 | 543 | 28 | 28 | 2694 | 80 | 81 |
| Men | 108 | 8 | 8 | 250 | 14 | 14 | 241 | 28 | 29 | 1538 | 95 | 102 |
| Women | 162 | 11 | 12 | 353 | 21 | 21 | 303 | 28 | 28 | 1155 | 65 | 65 |

* All cancer sites with the exception of non-melanoma skin cancer
