# Supplementary material for: Increasing Burden of Early-Onset Cancers: Disentangling the Contributions of Changes in Risk from Demographic Shifts
Source: Cancer Res Commun. 2026 Jul 1;6(7):1539–45. doi: 10.1158/2767-9764.CRC-26-0176 (PMC13319521; doi:10.1158/2767-9764.CRC-26-0176)
Supplement: Supplementary Figure 1 — Mean annual age-standardized (2013 European standard) incidence rates for all cancers (C00-43, C45-97), by age group (early- vs later-onset), sex, and 5-year period, 1982-2021, Switzerland. [file crc-26-0176_supplementary_figure_1_suppsf1.docx]

**Supplementary Figure 1 – Mean annual age-standardized (2013 European standard) incidence rates for all cancers (C00-43, C45-97), by age group (early- vs later-onset), sex, and 5-year period, 1982-2021, Switzerland.**

|  |  |  |
| --- | --- | --- |
| **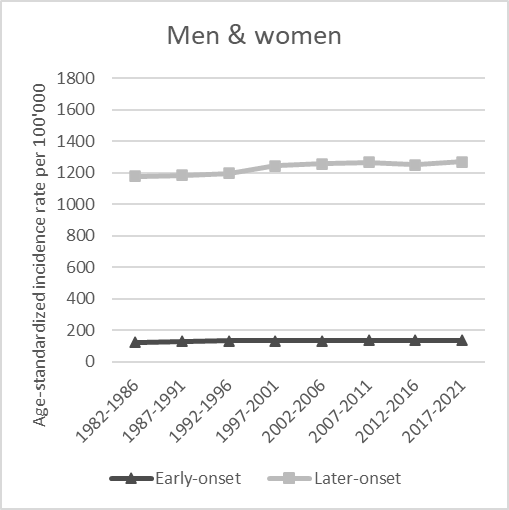** | **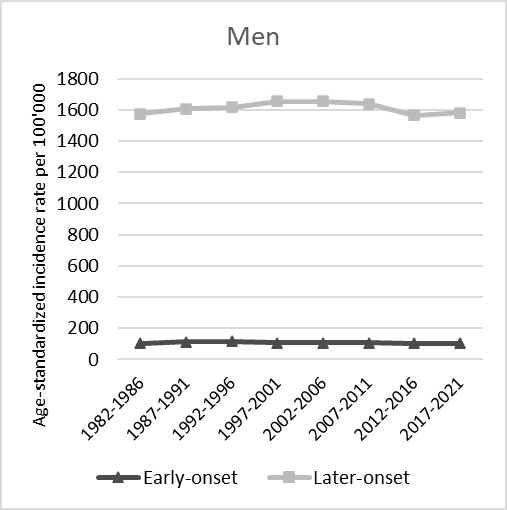** | **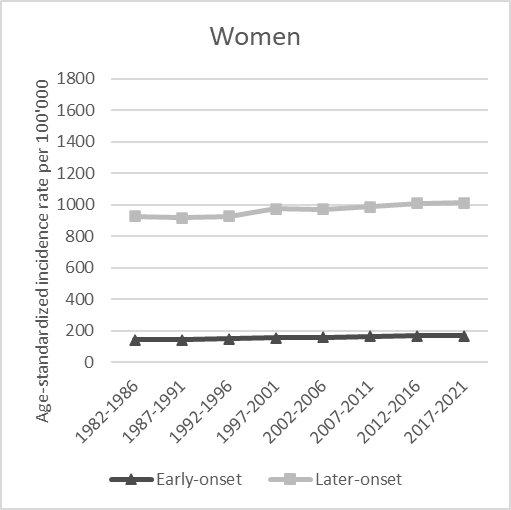** |
