# Supplementary material for: Increasing Burden of Early-Onset Cancers: Disentangling the Contributions of Changes in Risk from Demographic Shifts
Source: Cancer Res Commun. 2026 Jul 1;6(7):1539–45. doi: 10.1158/2767-9764.CRC-26-0176 (PMC13319521; doi:10.1158/2767-9764.CRC-26-0176)
Supplement: Supplementary Figure 3 — Mean annual age-standardized (2013 European standard) incidence rates among women, by main cancer sites (breast, colorectal, lung, skin melanoma) and age group (early- vs later-onset), and 5-year period, 1982-2021, Switzerland. [file crc-26-0176_supplementary_figure_3_suppsf3.docx]

**Supplementary Figure 3 – Mean annual age-standardized (2013 European standard) incidence rates among women, by main cancer sites (breast, colorectal, lung, skin melanoma) and age group (early- vs later-onset), and 5-year period, 1982-2021, Switzerland.**

| **Early-onset cancers** | | | |
| --- | --- | --- | --- |
| **Breast cancer (C50)** | **Colorectal cancer (C18-20)** | **Lung cancer (C33-34)** | **Skin melanoma (C43)** |
| 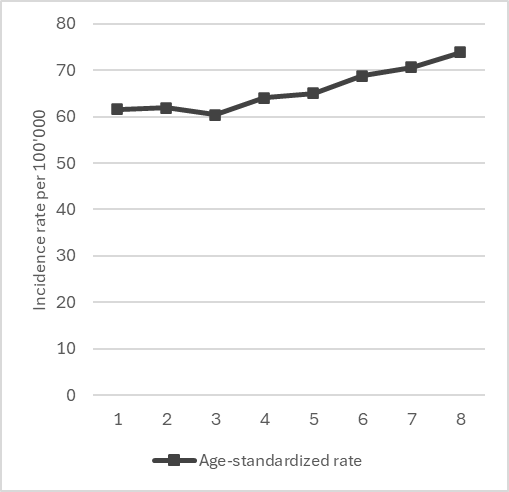 | 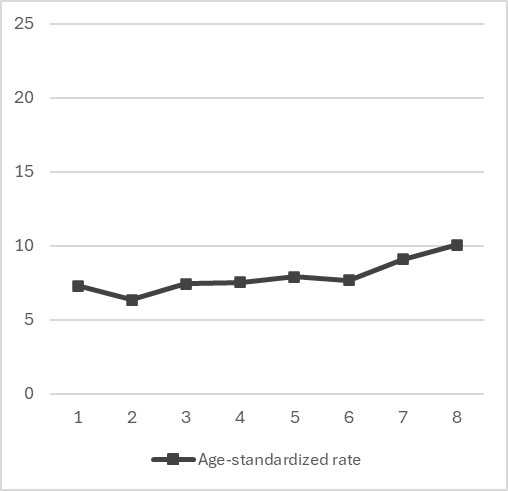 | 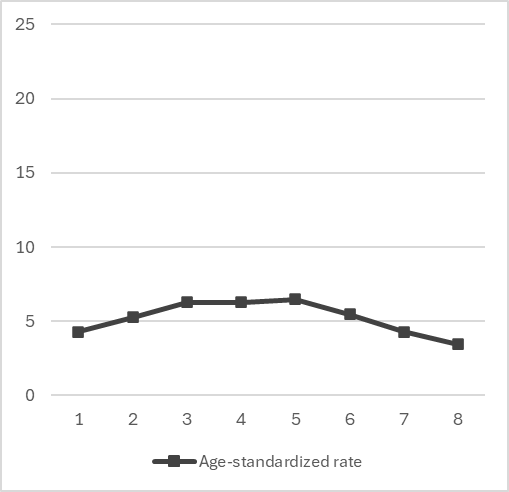 | 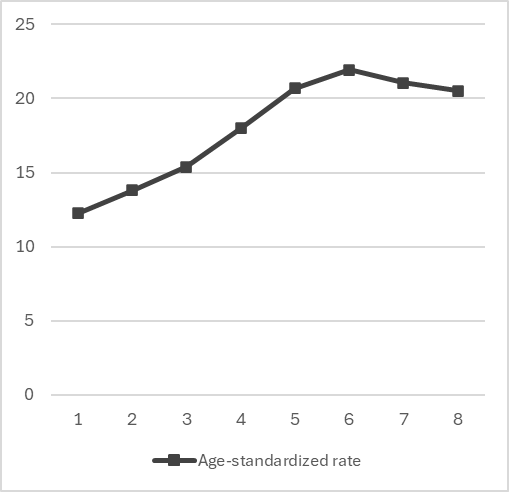 |
| **Later-onset cancers** | | | |
| 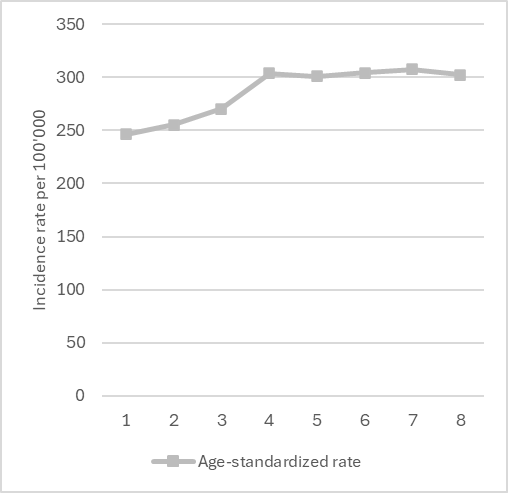 | 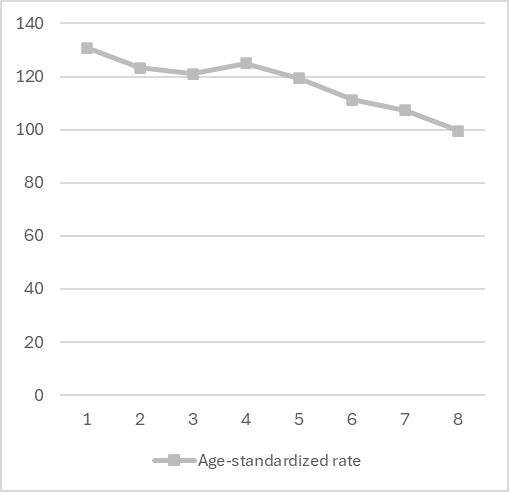 | 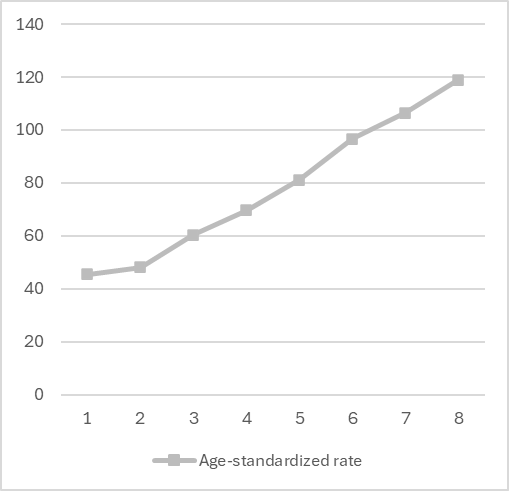 | 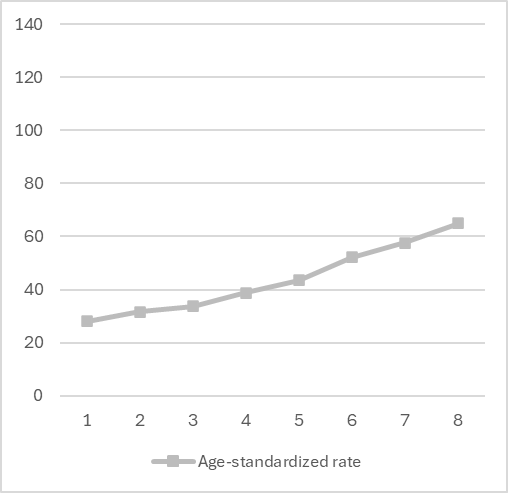 |
