# Supplementary material for: Increasing Burden of Early-Onset Cancers: Disentangling the Contributions of Changes in Risk from Demographic Shifts
Source: Cancer Res Commun. 2026 Jul 1;6(7):1539–45. doi: 10.1158/2767-9764.CRC-26-0176 (PMC13319521; doi:10.1158/2767-9764.CRC-26-0176)

**Supplementary Figure 4 – Proportion of each age subgroup (20-29, 30-39, 40-49) within the early-onset population, 1982-2021, Switzerland.**


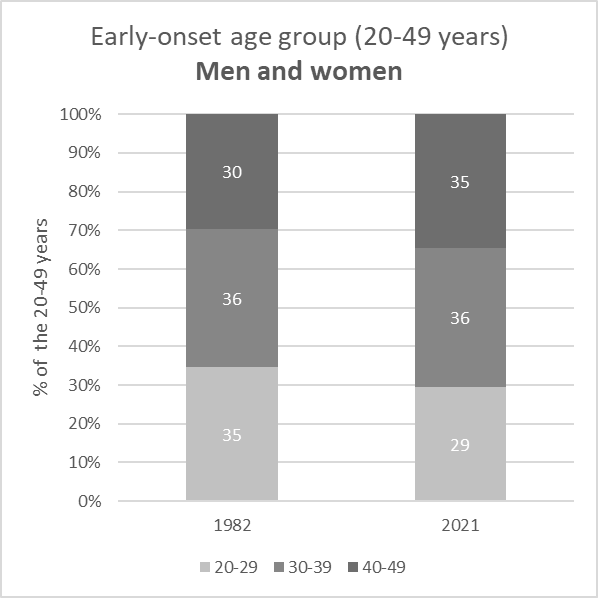

Supplement: Supplementary Figure 4 — Proportion of each age subgroup (20-29, 30-39, 40-49) within the early-onset population, 1982-2021, Switzerland. [file crc-26-0176_supplementary_figure_4_suppsf4.docx]
