# Supplementary material for: Increasing Burden of Early-Onset Cancers: Disentangling the Contributions of Changes in Risk from Demographic Shifts
Source: Cancer Res Commun. 2026 Jul 1;6(7):1539–45. doi: 10.1158/2767-9764.CRC-26-0176 (PMC13319521; doi:10.1158/2767-9764.CRC-26-0176)

**Supplementary Figure 5 – Cancer burden function. The cancer burden can be depicted as a function of demographic factors (that is, population growth and ageing) on the one hand and of cancer risk on the other hand. In our study, population growth and ageing determine *C_demographic_*, while exposure to carcinogenic factors, detection practices, and tumors classification determine *C_risk_*.**


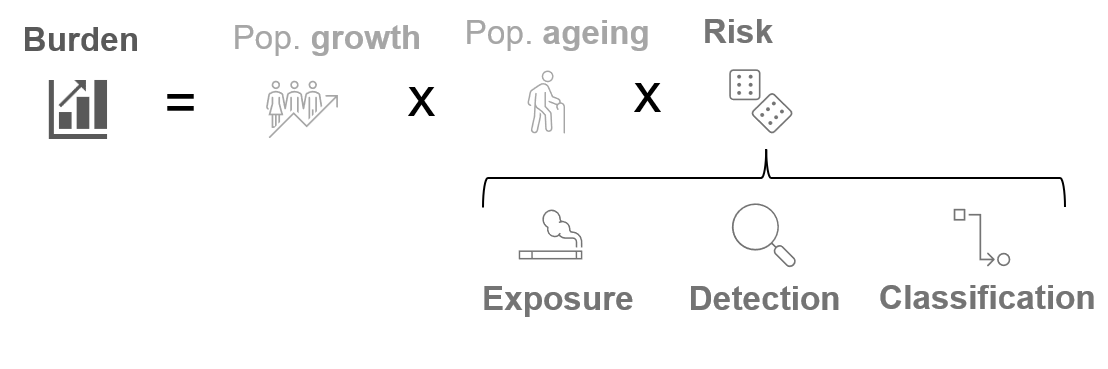

Supplement: Supplementary Figure 5 — Cancer burden function. The cancer burden can be depicted as a function of demographic factors (that is, population growth and ageing) on the one hand and of cancer risk on the other hand. In our study, population growth and ageing determine Cdemographic, while exposure to carcinogenic factors, detection practices, and tumors classification determine Crisk. [file crc-26-0176_supplementary_figure_5_suppsf5.docx]
